# Potential impacts of 2.3.4.4b highly pathogenic H5N1 avian influenza virus infection on Snow Goose (*Anser caerulescens*) movement ecology

**DOI:** 10.1371/journal.pone.0328149

**Published:** 2025-07-28

**Authors:** Jeffery D. Sullivan, Michael L. Casazza, Rebecca L. Poulson, Elliott L. Matchett, Cory T. Overton, Mike Carpenter, Austen A. Lorenz, Fiona McDuie, Michael Derico, Elizabeth W. Howerth, David E. Stallknecht, Diann J. Prosser

**Affiliations:** 1 U.S. Geological Survey, Eastern Ecological Science Center, Laurel, Maryland, United States of America; 2 U.S. Geological Survey, Western Ecological Research Center, Dixon Field Station, Dixon, California, United States of America; 3 Southeastern Cooperative Wildlife Disease Study, College of Veterinary Medicine, Department of Population Health, The University of Georgia, Athens, Georgia, United States of America; 4 U.S. Fish and Wildlife Service, Sacramento National Wildlife Refuge Complex, Willows, California, United States of America; 5 Department of Pathology, College of Veterinary Medicine, Department of Population Health, The University of Georgia, Athens, Georgia, United States of America; Hokkaido University: Hokkaido Daigaku, JAPAN

## Abstract

While wild waterfowl are known reservoirs of avian influenza viruses and facilitate the movement of these viruses, there are notable differences in the response to infection across species. This study explored differential responses to infection with highly pathogenic avian influenza in Snow Geese (*Anser caerulescens*) located in the California Central Valley. Though H5 antibody prevalence was high across years among birds sampled in the winter (75% in both years via hemagglutination inhibition), these values were even higher among birds sampled in summer that failed to migrate (i.e., August 2023 = 100% and August 2024 = 93% via hemagglutination inhibition). Birds that failed to migrate were also generally lighter than birds sampled in the winter and presented notable damage to cerebrum and cerebellum. In December 2022, a single individual positive for infection with H5N1 at the time of sampling indicated reduced movement during the 14 days following sampling but completed spring migration comparably with uninfected conspecifics. However, while no birds were actively infected during sampling and marking in 2023, two marked geese departed for migration late and one did not migrate at all. Additional banded birds marked in August have been reencountered in scenarios ranging from hunter harvest at a different site over a year later to found dead shortly after banding. Our data indicate that Snow Geese infected with HPAI have the potential to express variable outcomes following infection with highly pathogenic H5N1, ranging from rapid recovery within a migratory season to death. These data also suggest that the abnormal failure of some Snow Geese to migrate from the Central Valley is likely driven by HPAI infection.

## Introduction

Wild waterfowl are known reservoirs of avian influenza viruses (AIVs) and facilitate the movement of these viruses within a flyway [1,2], across flyways [3,4], and even between continents [5,6]. However, the potential for AIV dispersal can vary greatly by species susceptibility and behavior, migratory season, and virus strain. There are well documented differences in AIV infection prevalence between species, with dabbling ducks usually more likely to be infected than geese [7,8]. Similarly, challenge studies have indicated that susceptibility to infection, clinical signs of infection, and mortality all differ depending on viral strain and waterfowl species infected [9,10]. Differences in AIV infection prevalence in ducks also varies seasonally and is primarily associated with fall rather than spring migration [7,11].

Beyond differing prevalence rates and clinical responses, previous research has also indicated that infection with low pathogenic avian influenza (LPAI) can impact the movement ecology of wild waterfowl based on species. A study in California using telemetry to track waterfowl movement found that Northern Pintails (*Anas acuta*) and Canvasbacks (*Aythya valisineria*) infected with LPAI had significantly lower space use than non-infected individuals, but there was no difference between infected and non-infected Mallards (*Anas platyrhynchos*) [12] which aligns with similar observations in other work [13]. Similarly, species such as Bewick’s Swan (*Cygnus columbianus*) [14] and Canvasback that experienced LPAI infection delayed migration the following season, no such impact was observed with Northern Pintail [12] or Mallards [13]. Though antibodies conferred by surviving previous infection appear to modulate the impacts of recurrent infection even in wild settings [15].

While there are few studies that explore the impact of LPAI infection on movement and migration, even less is known about the impacts of highly pathogenic avian influenza (HPAI) infection on wild waterfowl outside of a laboratory setting. A study on Mallards from Tennessee, USA found that 11 Mallards infected with HPAI H5N1 clade 2.3.4.4b had no clear differences in local or migratory movement compared to non-infected conspecifics [16]. Similarly, a single White-faced Whistling Duck (*Dendrocygna viduata*) marked in Nigeria [17] and five Spot-billed Ducks (*Anas poecilorhyncha*) in South Korea [18] were found to complete migration following HPAI infection. Conversely, a single Lesser Scaup (*Aythya affinis*) from Maryland, USA, expressed restricted local movements compared to non-infected birds but died three days following marking [19] Given this paucity of information regarding how HPAI infection influences the movements of wild birds, obtaining telemetry data across as many species as could support the improvement of transmission risk models and the ability to adequately predict the potential implications of HPAI outbreaks on agriculture and wild bird populations.

During a telemetry study focused on the migratory ecology of Snow Geese in the California Central Valley, a single goose tested positive for infection with HPAI, allowing for the opportunity to follow this bird through the migratory period and compare behaviors to those of uninfected conspecifics. Additionally, following this initial detection, many geese, including some marked as part of the telemetry study, either departed for migration after a severe delay or failed to migrate from the wintering grounds altogether, behavior which had not previously been observed. Given the noted susceptibility of Snow Geese to HPAI in both laboratory studies [20] and wild mortality events [21,22] we sought to improve our understanding of infection outcomes in this species. Our objectives were to (1) characterize the movement of the infected individual relative to non-infected conspecifics marked on the same day and (2) to characterize movements of observed non-migrants and determine if they were or had been infected with highly pathogenic H5N1 or had pathological changes that may have affected their ability to migrate.

## Materials and methods

### Capture, sampling, and marking

#### Apparently healthy birds for telemetry study.

On 15 December 2022 and 7 March 2024 we captured Snow Geese, a species known to migrate between North America and Eurasia (more general ecology information available from [23]), on the Delevan National Wildlife Refuge (NWR), Colusa County, California (39.308°, −122.097°) using rocket nets [24]. All birds appeared healthy at the time of processing, with no apparent displays of illness or infection typical of waterfowl infected with HPAI [25]. Upon capture, geese were placed into large holding containers and kept out of direct sunlight until they could be processed. Birds were individually banded with U.S. Geological Survey, Bird Banding Laboratory metal bands and data on age and sex were recorded. A subset of birds was then selected (preference for adult females in 2022/23, no preference in 2023/24) for fitting with a transmitter. Selected individuals were marked with Ornitrack-N38 (inner diameter: 38 mm, outer diameter: 46–61 mm; 38g) or Ornitrack-N44 telemetry collars (inner diameter: 44 mm, outer diameter: 53–68 mm; 45g). Collars were programed to collect locational fixes every 15 minutes.

In addition to banding and marking, a cloacal and oropharyngeal swab [26] were collected from each bird using sterile polyester-tipped applicators and placed as paired samples (by bird) in vials containing 2 mL of cold virus transport medium (VTM) with the composition previously reported [19]. Swabs were stored on ice for <12 hours following collection and were shipped on ice to the laboratory and held at −80°C until processing. Additionally, up to 3 mL of blood was taken from the jugular vein (of birds not marked with transmitters in 2022 and all birds in 2024). Blood was placed in a BD Vacutainer SST (BD, Franklin Lakes, New Jersey, USA) and stored on ice for <12 hours. Blood samples were centrifuged for 20 minutes at 2500 rpm to separate serum.

Appropriate ethical review committee approval was received prior to the capture, sampling, and marking of any birds (USGS EESC ACUC 2022-11P; USGS WERC ACUC Review; and Federal banding permit #21142). All sampling of healthy birds required no use of anesthesia and was conducted as quickly as possible to reduce handling stress.

#### Sampling birds that failed to migrate.

Over the summers of 2023 and 2024, flocks of ~200 and ~1000 Snow Geese, respectively, uncharacteristically continued to reside on wetland ponds of the Sacramento NWR (39.418° N, 122.160° W), despite conspecifics initiating migration to breeding areas by late-March through mid-April (see Results). All remaining individuals appeared visually emaciated (exposed keel). A similar situation was observed in Yolo Bypass Wildlife Area (38.562° N, 121.629° W) when a flock of 120 Snow Geese failed to migrate in the summer of 2024. Thus, on 22 August 2023 and 8 August 2024, in an effort to assess potential causes for this behavior, we captured Snow Geese from the Sacramento NWR at night using an airboat and spotlighting techniques [27]. Similarly, Snow Geese were captured on 28 June 2024 at the Yolo Bypass Wildlife Area with rocket nets [28]. We collected swab and blood samples following the methods described above. In addition, two geese were collected via shotgun (i.e., immediate lethality, no anesthesia required) each summer (2023 from Sacramento NWR, 2024 from Yolo Bypass Wildlife Area) for necropsy evaluation at the Southeastern Cooperative Wildlife Disease Study. Carcasses were stored under refrigeration for six days prior to necropsy.

### Laboratory testing

For IAV detection, we extracted viral RNA from swab samples using the MagMAX-96 AI/ND Viral RNA Isolation Kit (Ambion/Applied Biosystems, Foster City, CA) and tested using real-time reverse transcriptase-PCR (rRT-PCR) targeting the IAV matrix (M) gene [29] Nucleic acids were also screened against primers specific for 2.3.4.4b H5 IAV in rRT-PCR; samples that yielded a cycle threshold value <40 were submitted to USDA National Veterinary Services Laboratory for official confirmation of subtype and pathogenicity following previously described methods [30]. IAV laboratory testing was completed after captured birds were released; hence, the infection status was not known at time of release.

For serology, serum samples were initially tested using a commercial blocking enzyme-linked immunosorbent assay (bELISA, IDEXX Laboratories, Westbrook, ME) for influenza A virus antibodies as described by the manufacturer. A serum-to-negative control (S/N) absorbance ratio < 0.7 was used as a positive cutoff threshold [31–33]. However, since manufacturer recommendations indicate a cutoff of < 0.5, we also report results using this value. All sera testing positive on bELISA were further tested for antibodies to H5 by hemagglutination inhibition (HI) and virus microneutralization (VN) as described by [34,35]. For both HI and VN H5 testing, two reverse genetics (rg) constructs were used as antigens. These included a low pathogenic virus with HA and NA from A/blue-winged teal/Texas/AI12–4150/2012 (H5N2) and remaining genes from A/Puerto Rico/8/1934 (PR8; H1N1) (this combination of low pathogenic H5 on the PR8 backbone is hereafter referred to as BWT). The clade 2.3.4.4b representative antigen [IDCDC-RG71A (H5N8)] consisted of HA and NA from A/Astrakhan/3212/2020 (H5N8) and remaining genes from PR8 (hereafter referred to as AST); the HA contains a modified protease cleavage site characteristic of low pathogenic IAV. Sera were also assessed for antibodies N1 using an enzyme-linked lectin assay (ELLA) as previously described, with A/ruddy turnstone/New Jersey/AI13–2948/2013(H10N1) used as antigen [34]. Positive threshold titers for HI, VN, and ELLA were 8, 20, and 80, respectively. For geometric mean titer (GMT) calculations, performed in the R statistical platform [36], antibody titers of <8 and ≥1024 (HI) and <20 and ≥ 2560 (VN) were transformed as 4 and 1024 for HI and 10 and 2560 for VN [37].

#### Pathology.

In addition to serologic testing, four nonmigrating geese were euthanized in August 2023 and July 2024. Euthanized individuals were randomly selected from the population. Cloacal and choanal swabs were collected and tested for influenza A by PCR. In addition, brain swabs and tissue were collected from the two 2024 birds for influenza PCR, as well as serum for influenza bELISA. Brains from the 2023 birds and heads, with calvarium removed, from the two 2024 birds were fixed in 10% buffered formalin. Heads were subsequently decalcified for further processing. Tissues from the four birds were routinely processed and embedded in paraffin and 4 micron sections were stained with hematoxylin and eosin (HE). Immunohistochemical staining for influenza A nucleoprotein and gial fibrillar acidic protein (GFAP; S1 Table) was performed on serial 4 m sections on an automated stainer (intelliPATH, Biocare).

### Movement data processing and analysis

Prior to analysis, telemetry data were reviewed to ensure all fixes were from live birds (i.e., removed fixes from dead birds or lost transmitters). The 2022/23 dataset was further reduced to include only females (only one marked male in this dataset) while the 2023/24 dataset was not subset by age or sex. After filtering, we characterized local movements of each marked bird with complete data during the 14 days following marking (beginning the day after release). We focused on the first fourteen days following release (beginning the day after release) as this is the window traditionally used to represent the infectious period of HPAI infected waterfowl [19,38]. While infectious period can vary dramatically between species [39,40], the use of a broader window allows further certainty that any unusual behaviors during the actual infectious period would be captured. We calculated two metrics to assess local movements: the daily distance moved and the daily minimum convex polygon (MCP) area. Daily distance moved was calculated by finding the distance between each consecutive locational fix within a calendar day and summing this distance for each individual with at least 72 fixes (75% of possible fixes within a given day). Distance between fixes was calculated within R Studio [41] using the distHaversine function [42]. Daily MCP area was found by using the “mcp” function [43] to generate shapefiles of the 100% daily MCP, and the area of these shapefiles was calculated in ArcMap 10.8.1 [44].

In addition to characterizing local movements, we also identified contact events between the marked infected bird and other marked birds during this initial 14-day window. A contact event was defined as any fix within 25 m and 15 min of a fix from the infected individual [19]. These buffers were used since individual transmitters were turned on at different times, depending on time of marking, hence although all transmitters are set to the same fix cycle, the fixes are not taken at the same time.

Finally, we sought to characterize migratory movements of all individuals for which a full migratory track was available via three metrics: migration initiation date, migration end date, and total migration duration. Migration initiation was identified by manually reviewing data and locating the first fix representing sustained movement away from the wintering grounds [45]. Similarly, migration end date was selected as the first point from which there were repeated locational fixes with limited displacement during the breeding period [46]. Migration duration was calculated as the number of whole days from migration initiation to migration end date. To ensure comparability in migration metrics we separated individuals based upon not only infection status (2023 birds) or migration timing (2024 birds), but also final migratory end point.

Data supporting all results in this manuscript are available from [47].

## Results

### Apparently healthy birds

A total of 66 apparently healthy Snow Geese were captured and sampled on 15 December 2022 and 7 March 2024, with laboratory processing detecting only two active infections with AIV, both of which were HPAI infections from the December 2022 sampling event (Table 1). However, no isolates or sequences were obtained from these positive samples. Conversely, antibody prevalence was high across both sampling years, with at least 75% of birds in each year positive at the S/N < 0.7 threshold and more than 75 and 63% of birds in each year possessing H5 antibodies as detected via HI and VN, respectively (Table 2).

**Table 1:** Prevalence (representing active infection) of influenza A virus for wild Snow Geese (*Anser caerulescens*) sampled on wintering areas at Delevan and Sacramento National Wildlife Refuges and Yolo Bypass Wildlife Area in California, USA. Prevalence identified using 2.3.4.4 HP H5 rRT-PCR (M + , Ct values ≤ 40).

Sampling Date	Location	Health Status	Age	Sex	Weight (g)^^^		Active Infection	
					n	$\overset{―}{x}$	n	H5 + Prev
12/15/2022	Delevan NWR	Apparently Healthy	Cumulative		50	2155.3	50	4%
			AHY	M	11	2298.7	11	0%
			AHY	F	33	2147.5	33	3%^*^
			HY	M	4	2015.5	4	0%
			HY	F	2	1774.0	2	50%
8/22/2023	Sacramento NWR	Non-Migrants	Cumulative		27	1638.5	27	0%
			ASY	M	15	1680.7	15	0%^**^
			ASY	F	5	1627.8	5	0%
			SY	M	3	1508.0	3	0%
			SY	F	4	1591.5	4	0%
3/7/2024	Delevan NWR	Apparently Healthy	Cumulative		16	1903.5	16	0%
			ASY	M	3	2093.3	3	0%
			ASY	F	1	1736.0	1	0%
			SY	M	4	2006.0	4	0%^+^
			SY	F	8	1802.0	8	0%^++^
6/28/2024	Yolo Bypass	Non-Migrants	Cumulative		14	2074.9	14	0%
			ASY	M	8	2091.0	8	0%
			ASY	F	6	2053.5	6	0%
8/8/2024	Sacramento NWR	Non-Migrants	Cumulative		15	1513.3	15	0%
			ASY	M	5	1439.2	5	0%^**^
			ASY	F	10	1550.4	10	0%

^ All birds were measured to the nearest gram except for individuals captured at the Yolo Area Wildlife Bypass on 6/28/2024 which could only be measured up to 2100 g. Any birds over 2100 g were treated as 2100 g for this analysis.

* Includes the single infected transmittered bird

** Includes a single individual that initially tested positive via PCR but which was not confirmed by NVSL

+ Includes two individuals that initiated migration later than normal for this species.

++ Includes one individual that never departed on spring migration.

**Table 2:** Detection of antibodies to influenza A virus (bELISA) as well as H5 (Hemagglutination inhibition and Virus neutralization with cutoffs of ≥8 and ≥20, respectively) and N1 (Enzyme-linked lectin assay, cutoff ≥80) specifically in wild Snow Geese (*Anser caerulescens*) sampled on wintering areas at Delevan (sampled 12/15/2022 and 3/7/2004) and Sacramento National Wildlife Refuges (8/22/2023 and 8/8/2024) and Yolo Bypass Wildlife Area (6/28/2024) in California, USA. When including any values with a numeric operator in calculating a geometric mean the next closest dilution value was used (i.e., < 8 became 4 but ≥2560 became 2560).

		bELISA		Hemagglutination inhibition				Virus neutralization				Enzyme-linked lectin assay	
Sampling Date	n	% Positive (S/N < 0.5)	% Positive (S/N < 0.7)	AST log2 $\overset{―}{x}$	% Positive	BWT log2 $\overset{―}{x}$	% Positive	AST log2 $\overset{―}{x}$	% Positive	BWT log2 $\overset{―}{x}$	% Positive	ELLA log2 $\overset{―}{x}$	% Positive
12/15/2022	16	75%	88%	3.69	75%	3.44	38%	6.26	63%	5.26	56%	8.38	75%
8/22/2023	27	96%	100%	5.07	100%	3.89	85%	8.84	100%	5.21	70%	10.40	100%
3/7/2024	16	75%	75%	4.38	75%	3.25	44%	7.88	69%	4.63	50%	8.20	75%
6/28/2024	14	57%	86%	4.00	93%	2.21	21%	9.61	100%	4.11	56%	9.61	100%
8/8/2024	14	93%	93%	4.50	86%	3.57	71%	7.75	86%	3.68	21%	8.61	93%

**Fig 1:**
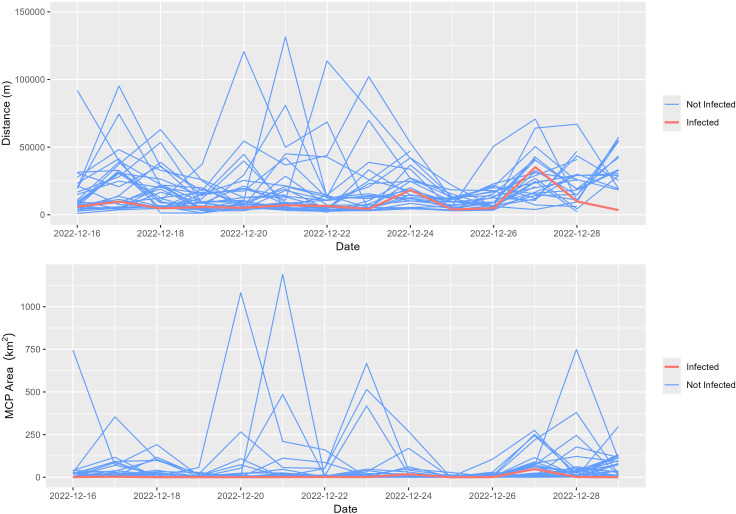
Daily distance moved and daily minimum convex polygon (MCP) area for Snow Geese (*Anser caerulescens*) marked with GPS-GSM transmitters on a wintering area at Delevan National Wildlife Refuge (California, USA) on 15 Dec 2022. Daily distance moved was calculated by finding the distance between each consecutive locational fix within a calendar day and summing this distance for each individual with at least 72 fixes (75% of possible fixes within a given day). The red line represents data from a single Snow Goose that was later revealed to have been naturally infected with HPAI and shedding virus at the time of marking.

In 2023, a total of 30 marked individuals (29 non-infected, 1 infected) transmitted data for the duration of the first 14 days following capture, sampling, and release. During this initial window non-infected Snow Geese had average daily movement distances ranging from 7448–45384 m, while the lone infected bird had daily movements ranging from 3434–35240 m and an average daily movement distance of 8910 m, placing it at the 10^th^ percentile (Fig 1). A similar trend was observed with daily MCP area, which ranged from < 1–1189 km2 among non-infected birds, with an average of 40.5 km^2^. However, the single infected bird had a daily MCP ranging from < 1–48.1 km^2^ with an average of 5.74 km^2^, placing it in the 13^th^ percentile. We observed a total of 21 contacts between the infected bird and 12 unique non-infected birds, ranging from a single fix to 12 hours of continued proximity.

**Fig 2:**
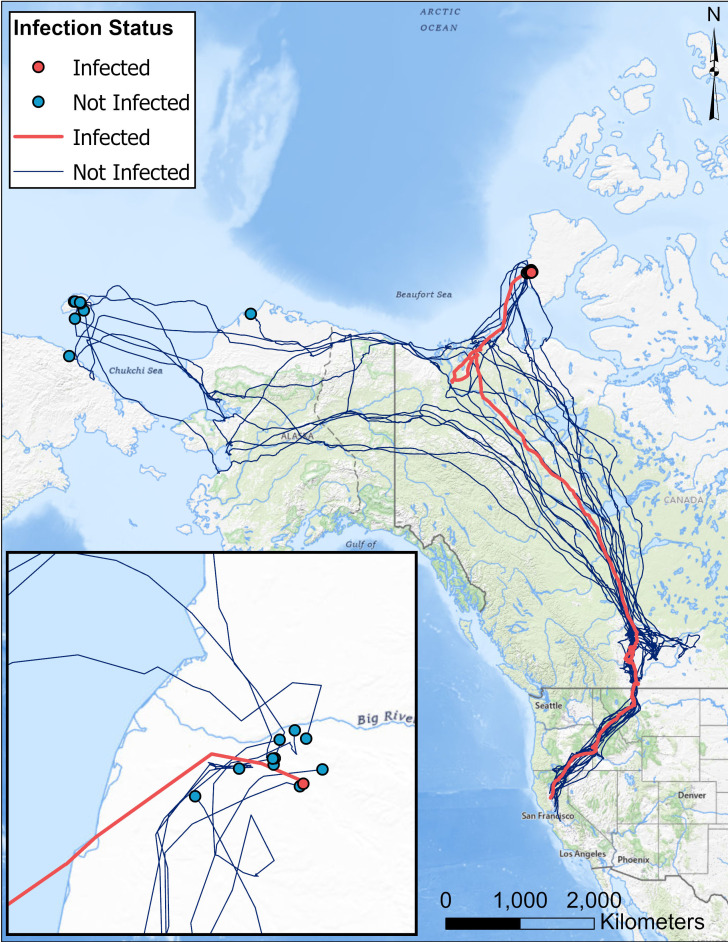
Spring migratory paths for Snow Geese (*Anser caerulescens*) marked with GPS-GSM transmitters on a wintering area at Delevan National Wildlife Refuge (California, USA) on 15 Dec 2022. Lines indicate migratory pathways while dots indicate the first location at the likely breeding site. The inset map shows the proximity of the identified likely breeding site of a single Snow Goose that was later revealed to have been infected with HPAI at the time of marking (red) relative to individuals that test negative for HPAI (blue). Basemaps are taken from the USGS National Map.

In 2023, a total of 19 individuals (18 non-infected, 1 infected) transmitted data through spring migration. Snow Geese migrated primarily to Wrangel Island (Russia) and Banks Island (Canada), with the infected bird migrating to Banks Island in the Canadian portion of the Artic Archipelago. When compared only to individuals migrating to the same breeding site, the infected bird was in the 36^th^ percentile for migration initiation date, 27^th^ percentile for arrival date, and 36^th^ percentile for migration duration (Table 3). While there may be minor differences in the migration timing and duration between sites, Snow Geese, including the infected bird, appeared to take the same general migratory route until reaching stopover sites in southern Alberta, Canada (Fig 2).

**Table 3:** Average spring migration initiation and arrival dates on breeding grounds for Snow Geese (*Anser caerulescens*) marked with GPS-GSM transmitters on wintering areas at Delevan National Wildlife Refuge (Califonria, USA) on 15 Dec 2022 and 7 Mar 2024 based on IAV infection status from testing conducted at the time of marking and migration initiation dates, respectively. All birds marked in 2024 were negative for active infection with IAV at the time of marking. Brackets indicate the minimum and maximum values observed in a category.

Year	Bird Status	Migration Endpoint	n	Initiation Date	End Date	Duration (days)
2023	Infected	Banks Island	1	3/17/2023	5/29/2023	73
	Not Infected	Banks Island	10	3/19/2023 [3/03-4/04]	5/30/2023 [5/28-6/4]	73 [55-89]
	Not Infected	Wrangel Island	6	3/22/2023 [3/7-3/31]	5/27/2023 [5/21-6/1]	66 [55-86]
	Not Infected	Leningradsky	1	4/17/2023	6/6/2023	50
	Not Infected	Peard Bay	1	3/7/2023	5/24/2023	78
2024	Normal Migrant	Banks Island	7	4/5/2024 [3/25-4/26]	5/31/2024 [5/27-6/9]	57 [44-66]
	Late Migrant	Banks Island	1	5/12/2024	6/8/2024	27
	Normal Migrant	Prince Alberts Island	1	4/24/2024	5/30/2024	36
	Late Migrant	Prince Alberts Island	1	5/26/2024	6/8/2024	13
	Normal Migrant	Wrangel Island	1	3/28/2024	5/29/2024	63
	Normal Migrant	Peard Bay	1	4/9/2024	5/31/2024	52

### Delayed and non-migrants

An additional 56 samples were collected on 22 August 2023, 28 June 2024, and 8 August 2024 from Snow Geese that failed to migrate during normal timelines. For birds sampled on the Delevan and Sacramento NWRs, body weight was notably higher in early sampled birds than in late sampled birds (Table 1). However, birds sampled at Yolo Bypass Wildlife Area had higher masses than other birds that failed to migrate, though qualitative visual observations indicated that these birds appeared to lose mass over time and behaved unusually. For instance, when the rocket net was fired, an event causing considerable noise and disruption, individuals beyond the range of the net swam slowly away instead of flying despite capture occurring among flight-capable individuals and prior to wing molt.

While we detected only a single active infection from these individuals (Table 1), the rate of prior infections was slightly higher among the birds in flocks that did not migrate at normal times of year than for the apparently healthy birds (Table 2). For instance, every individual sampled at the Sacramento NWR in August 2023 tested positive for antibodies to influenza A virus compared to 88% in December. Cumulative results from subtyping of previous infections with both HI and VN indicated 26 of 32 apparently healthy birds, and all but one individual that failed to migrate by August showed antibodies to H5 and N1. The high antibody levels for AST relative to BWT for individuals that failed to migrate indicate likely previous infection with highly pathogenic H5N1, such levels were much more comparable in the birds sampled while apparently healthy.

**Fig 3:**
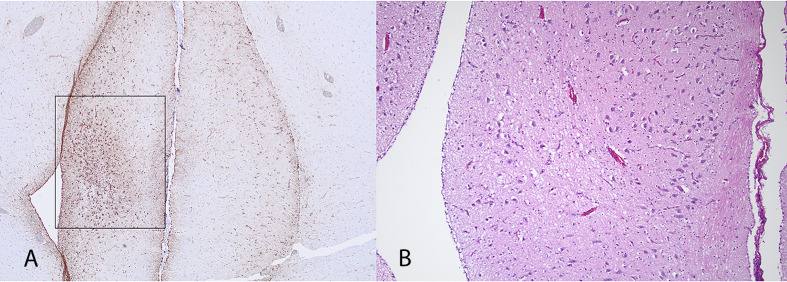
Unilateral focus of damage in the septum pellucidum of one of the 2023 snow geese. **A)** GFAP immunohistochemistry. Note the large amount of dark brown staining in the black box which indicates increased numbers of astocytes (astrogliosis) as opposed to the opposite side. DAB chromogen. **B)** HE staining of the same focus characterized by mild vacuolation, loss of neurons, and prominent capillaries lined by plump endothelium.

**Fig 4:**
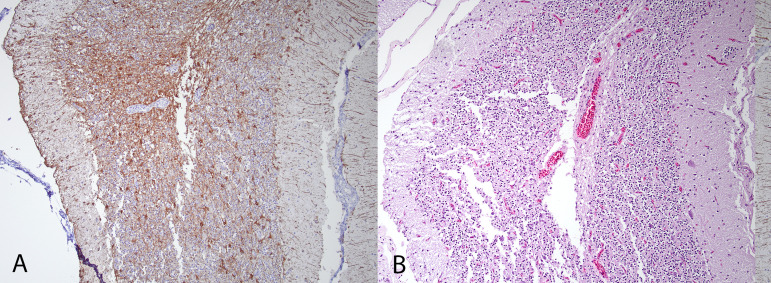
Cerebellar folium of one of the 2024 geese. **A)** GFAP immunohistochemistry. Severe astrogliosis characterized by the large amount of brown staining in the molecular layer and internal granular. DAB chromogen. **B)** HE staining of the same folium. The molecular layer is thin and rarified with decreased Purkinje cells and internal granular layer neurons.

Cloacal, choanal, and brain samples collected from the four nonmigrating birds tested negative for influenza A by PCR. Brain tissue from all birds also tested negative for influenza A by immunohistochemistry. Both birds sampled in 2024 tested positive for antibodies to AIV, H5, and N1. Serum was not available for the birds collected 2023. All birds had multiple asymmetric foci of astrogliosis detected by immunohistochemistry for GFAP in both cerebrum and cerebellum, which is a reactive change to previous damage. On HE stained sections, these foci corresponded to mild neuroparenchymal vacuolation, loss of neurons, and prominent capillaries in the cerebrum (Fig 3) and more severe changes in the cerebellum involving one or more folia. Affected folia had thinning and rarefaction of the molecular layer, decreased neuronal cellularity of the internal granular layer and vacuolation of the associated foliar white matter (Fig 4). We presume these changes to be the result of previous infection with influenza as these changes mirror the typical immunohistologic distribution of influenza virus antigen we previously observed in acutely infected snow geese.

**Fig 5:**
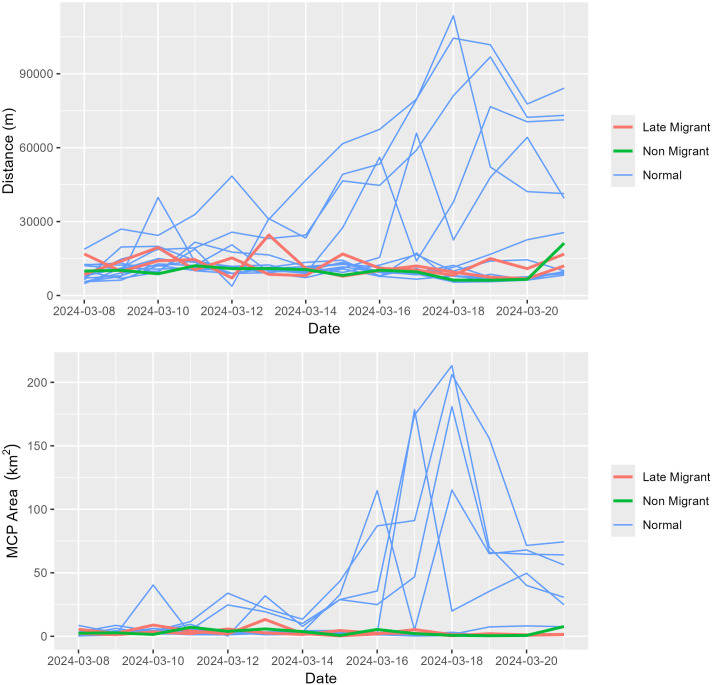
Daily distance moved and daily minimum convex polygon (MCP) area for Snow Geese (*Anser caerulescens*) marked with GPS-GSM transmitters on a wintering area at Delevan National Wildlife Refuge (California, USA) on 7 March 2024. Daily distance moved was calculated by finding the distance between each consecutive locational fix within a calendar day and summing this distance for each individual with at least 72 fixes (75% of possible fixes within a given day).

In 2024, a total of 16 individuals (13 normal migrants, 2 late migrants, and 1 non-migrant) transmitted data for the duration of the first 14 days following capture, sampling, and release. During this initial window, average daily movement distances ranged from 9699–57578 m and from11692–12393 m for normal migrants and late migrants, respectively. The non-migrant bird had daily movement distances ranging from 6204–21266 m. Average daily movement distances were 21763 m, 12042 m, and 10126 m (Fig 5) for normal migrants, late migrants, and the non-migrant, respectively. A similar trend was observed with daily MCP area (Fig 5). Interestingly, there have been 6 reported reencounters of the birds sampled and banded in Sacramento NWR on 22 August 2023 that failed to migrate within the traditional window for this species. Three individuals were found freshly dead on the Sacramento NWR (two encountered on 11 September 2023, and one encountered 14 November 2023) with a fourth found as skeletal remains on 24 February 2024. However, two individuals were later reencountered via hunter harvest, with the first bird harvested near Colusa, California (~23 km from banding location) on 15 December 2023 and the second near Stockton, California (~175 km from banding location) on 26 October 2024.

**Fig 6:**
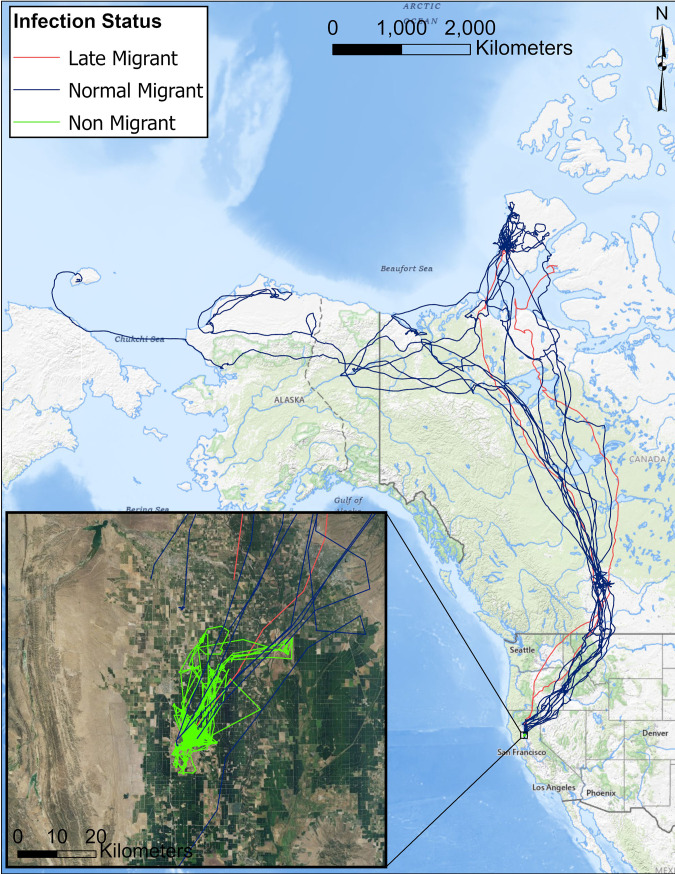
Spring migratory paths for Snow Geese (*Anser caerulescens*) marked with GPS-GSM transmitters on a wintering area at Delevan National Wildlife Refuge (California, USA) on 7 Mar 2024. Lines indicate all movements from the initiation of migration through 15 June 2024 for all birds that migrated during a normal migratory window (blue), and those that migrated with a late departure (red). All movements from marking through 15 June 2024 for the non-migrating bird are shown in the inset map (green). Basemaps are taken from the USGS National Map.

In 2024, a total of 12 individuals transmitted data through spring migration. As observed in 2023, Snow Geese primarily migrated to Banks Island, though birds were also observed migrating to Wrangel Island, and Peard Bay (Table 3). However, we also observed birds (marked as hatch year so second year at time of migration) that migrated directly to molting grounds in eastern Banks Island by traveling through Alberts Island. The two late migrants appeared to follow routes similar to those used by other birds that departed for migration at traditional times (Fig 6). It should be noted that comparisons between 2023 and 2024 should be limited due to differences in sex and age of marked individuals (i.e., 2023 included only adult females while 2024 included males and hatch year birds) as well as anecdotal reports of heavy snow on the breeding grounds and compact dense late-season sea-ice near Wrangel Island [48] which is indicative of weather conditions that limit nesting attempts.

## Discussion

Our data indicate that Snow Geese infected with HPAI have the potential to express variable outcomes, ranging from rapid recovery within a migratory season to death. For instance, one telemetry marked bird that was infected with HP H5N1 completed a normal migration while other individuals that had previously been infected with HP H5N1 based on serologic results suffered delayed migration or did not migrate at all. The long-term outcome of these nonmigrating geese is not currently clear. While some local mortality was observed, a single bird believed not to have migrated in 2023 was harvested approximately 16 months later 175 kilometers away from the point of capture. The differences in the response to HP H5N1 infection observed between Snow Geese may have been driven by a variety of factors including preexisting immunity to LP AIV [49] which is known to circulate in wild Snow Geese [7,50], variations in the specific HPAI genotype [51], differential exposure to contaminants [52] or other factors that would weaken immune response. Additional research could support parsing out these complex inter-relations.

The recovery and completion of subsequent migration by a Snow Goose infected with HPAI reported here is comparable to other species such as Mallards [16], White-faced Whistling Duck [17] and Spot-billed Ducks [18], which have all been observed to successfully migrate following HPAI infection. While sample size is limited for this observation, and the majority of previous similar reported instances, it does provide further insight into the long-term impacts of HPAI infection on at least some members of these populations that can be used to inform more complex modeling efforts [53]. Notably, the infected individual also followed the same migratory route to reach a stopover habitat in southern Alberta as all other marked birds in this study did, regardless of their migratory endpoint. Thus, there appears to be potential for relay transmission of infections on wintering grounds to reach different breeding grounds [54].

While the single actively HPAI-infected Snow Goose marked with a transmitter appeared to have comparable migratory ecology with non-infected conspecifics, this does not mean the infected individual did not express any impacts from infection. Instead, the infected Snow Goose had notably lower daily movements and overall space use compared to non-infected geese. This supports similar observations with species such as Northern Pintail and Canvasbacks which showed decreased space use during the period of active infection with LPAI [12]. Response to infection does appear highly species-specific as Mallards showed no such response to HPAI infection [16]

While infection with HPAI appears to have had no or limited impact on the migration of one tracked bird, the same cannot be said for the Snow Geese sampled in August which were visibly emaciated and failed to leave this wintering site for spring migration. While we cannot say for certain that the brain lesions observed in these individuals were caused by the HPAI infections, this does appear likely and has been reported in other species [55,56]. Similarly, weight loss is commonly associated with AIV infection in both challenge studies and wild waterfowl [49,57,58]. However, there is some dispute whether low body weight in infected wild waterfowl is a result of infection or just correlated with birds in poor condition that were already at high risk of infection [59,60]. It is possible that the low body weight observed in the non-migrating birds could also have resulted from behavior changes associated with brain injury occurring during previous HP H5N1 infection. Still, the tagging of three individuals that were already seropositive and later failed to migrate at normal times suggests that whatever response to infection led to this altered behavior built over time, as birds appeared healthy at the time of marking.

The data described in this manuscript provide unique insight into the impacts of HPAI infection on wild Snow Geese. The observed range in responses from reduced localized movement followed by apparent recovery and successful migration to long-term impacts resulting in a missed breeding season also demonstrates a knowledge gap in the available literature surrounding how individual species respond to infection and the factors that drive individual infection outcomes. While our sample size of tracked infected birds is insufficient for more detailed analysis, these results still provide useful insight and help to demonstrate the value of pairing disease sampling with ecology-focused animal movement studies.

## Supporting information

S1 Table Antibodies with host, source, retrieval information, and dilution. (DOCX)
