# Supplementary material for: Potential impacts of 2.3.4.4b highly pathogenic H5N1 avian influenza virus infection on Snow Goose (Anser caerulescens) movement ecology
Source: PLoS One. 2025 Jul 28;20(7):e0328149. doi: 10.1371/journal.pone.0328149 (PMC12303340; doi:10.1371/journal.pone.0328149)
Supplement: S1 Table — (DOCX) [file pone.0328149.s001.docx]

**Supporting Information for:**

Potential impacts of 2.3.4.4b highly pathogenic H5N1 avian influenza virus infection on Snow Goose (*Anser caerulescens*) movement ecology

Jeffery D. Sullivan^1^, Michael L. Casazza^2^, Rebecca L. Poulson^3^, Elliott L. Matchett^2^, Cory T. Overton^2^, Mike Carpenter^2^, Austen A. Lorenz^2^, Fiona McDuie^2^, Michael Derico^4^, Elizabeth W. Howerth^5^, David E. Stallknecht^3,^*, Diann J. Prosser^1^,*

^1^U.S. Geological Survey, Eastern Ecological Science Center, Laurel, MD, United States of America

^2^U.S. Geological Survey, Western Ecological Research Center, Dixon Field Station, Dixon, CA, United States of America

^3^Southeastern Cooperative Wildlife Disease Study, College of Veterinary Medicine, Department of Population Health, The University of Georgia, Athens, GA, United States of America

^4^ U.S. Fish and Wildlife Service, Sacramento National Wildlife Refuge Complex, Willows, CA, United States of America

^5^ Department of Pathology, College of Veterinary Medicine, Department of Population Health, The University of Georgia, Athens, GA, United States of America

*Co-Corresponding Authors

Email: [dprosser@usgs.gov](mailto:dprosser@usgs.gov) and [dstall@uga.edu](mailto:dstall@uga.edu)

**S1 Table. Antibodies with host, source, retrieval information, and dilution.**

| Antibody | Host | Source/Clone | Antigen retrieval | Dilution |
| --- | --- | --- | --- | --- |
| Influenza A | Goat | Meridian Life Science B65141G | Proteinase K | 1.0833 |
| GFAP | Mouse | Biogenex MU020-UC/GA-5 | Citrate pH 6 | 2.8194 |
